# Recurrent arthrocele and sterile sinus tract formation due to ceramic wear as a differential diagnosis of periprosthetic joint infection — a case report

**DOI:** 10.1080/17453674.2019.1616997

**Published:** 2019-05-16

**Authors:** Nico Maximilian Jandl, Tim Rolvien, Daniel Gätjen, Anika Jonitz-Heincke, Armin Springer, Veit Krenn, Rainer Bader, Wolfgang Rüther

**Affiliations:** a Department of Orthopedics, University Medical Center Hamburg-Eppendorf, Hamburg, Germany;; b Orthopedic University Hospital Bad Bramstedt;;; c Department of Osteology and Biomechanics, University Medical Center Hamburg-Eppendorf, Hamburg;; d Department of Orthopedics, Biomechanics and Implant Technology Research Laboratory, Rostock University Medical Center, Rostock;; e Medical Biology and Electron Microscopy Center, Rostock University Medical Center, Rostock;; f MVZ-Zentrum für Histologie, Zytologie und Molekulare Diagnostik, Trier, Germany

A 63-year-old female patient with total hip arthroplasty (THA) presented at our clinic with a massive swelling of the right hip joint. 13 years ago, cementless THA with an alumina ceramic-on-ceramic bearing (Biolox forte, CeramTec GmbH, Plochingen, Germany) had been performed due to advanced osteoarthritis.

In the first years after THA, the patient had been symptom-free. The patient then complained of a swelling when sitting. MRI and CT showed a fluid-filled tumor of the right hip joint expanding into the gluteal muscle. Laboratory infection parameters were normal (C-reactive protein: not detectable; leucocytes: 7.1 G/L).

The patient was twice bursectomized elsewhere 12 years after the THA and histopathological examination pointed to a granulomatous disease. Various differential diagnoses such as rheumatoid arthritis, sarcoidosis, tuberculosis and rare causes such as brucellosis, toxoplasmosis, echinococcosis and mycosis as well as an adverse local tissue reaction (ALTR) due to abrasive wear particles were considered.

As further diagnostic procedures including QuantiFERON test and chest radiography showed no signs of tuberculosis and sarcoidosis, we performed an open synovial biopsy 10 months later to search for abrasive wear particles and to exclude periprosthetic joint infection (PJI). The dorsal hip joint capsule and the arthrocele were completely resected. Histology showed granulomas of the foreign body type and very sparse birefringent wear particles. In 5 of 8 tissue samples, the histological criteria for PJI according to Krenn et al. (20179) were not met. The microbiological culturing of tissue samples and synovial fluid over 14 days remained sterile. As the arthrocele recurred, revision THA was performed another 2 months later with suspicion of ALTR to wear particles and the acetabular cup, which was firmly integrated into the bone, was exchanged (Allofit IT, Zimmer, Warsaw, IN, USA). No macroscopic abnormalities of the head–neck junction or the connection between ceramic liner and acetabular cup were observed. A new aluminum-zirconium composite ceramic head and acetabular liner (Biolox delta, CeramTec GmbH) was implanted. Particle analysis of tissue samples by CytoViva dark-field microscopy (CytoViva Inc, Auburn, AL, USA) indicated intracellular particles (Figure 1).

**Figure 1.**
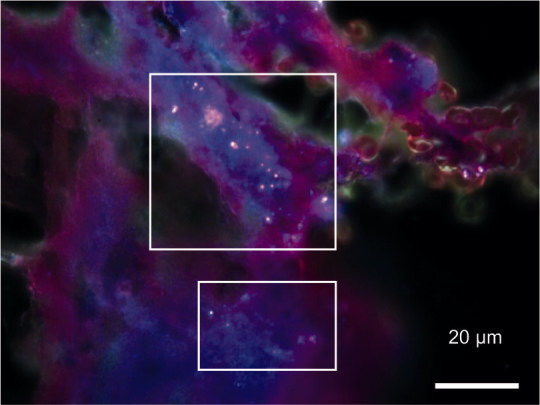
CytoViva dark-field microscopy (100×; CytoViva Inc, Auburn, AL, USA) of synovial tissue showed intracellular particles shining pink. The synovial tissue had been taken before the acetabular cup as well as the ceramic head and liner were removed.

6 months later, the patient presented with a reddened distal wound margin but no infection parameters. A large arthrocele was palpable, which finally emptied itself via a sinus tract, i.e., a major criterion for PJI according to the Musculoskeletal Infection Society (MSIS, Parvizi et al. 2011) was fulfilled. During 2-step revision surgery with total explantation of all components and subsequent spacer implantation, brownish-reddish fluid with semolina-like admixtures, but no purulence, was found periarticularly. Microbiology remained negative once again, but calculated antibiotic treatment was initiated. According to the histopathological synovial-like interface membrane (SLIM) consensus classification, the tissue morphology was between an infectious type (SLIM type II) and an adverse reaction (SLIM type VI) (Krenn et al. 2014, 2016, Perino et al. 2018), evaluated by 2 independent pathologists (Figure 2).

**Figure 2.**
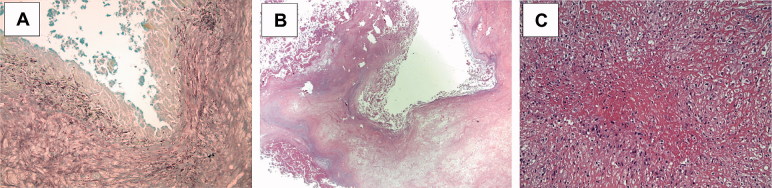
Histological view of the synovial tissue biopsies from the interface (synovia-like interface membrane, SLIM) of the THA revision 1 year after acetabular cup, ceramic head, and liner exchange. Grocott staining (100×) showing a pseudocyst with no evidence of mycosis (A). HE staining (12.5×) showing a fibrous cyst (B). HE staining (220×) showing a fibrinoid necrosis with focal palisading of fibroblasts and accumulation of macrophages (C).

As the wound secretion did not improve in 1 month, a Girdlestone procedure was done. All tissue and synovial samples remained sterile again and further special analysis concerning echinococcosis, toxoplasmosis, tuberculosis (microscopic analysis, cultures with 8 weeks of incubation and mycobacterial PCR), brucellosis and mycosis as well as a non-routine pan-bacterial PCR (bacterial and mycotic DNA) also remained negative. Apart from unchanged histopathological findings no other MSIS minor criteria were fulfilled, i.e., PJI was unlikely and antibiotic therapy was terminated as wound secretion did not improve postoperatively. MRI showed retained fluid draining via a sinus tract that was in direct connection with the hip joint. An autoimmune reaction to wear particles was our remaining explanation for the recurrent arthrocele formation. Since serum metal ion concentrations were in the reference range (chromium < 0.5 µg/L, cobalt < 0.9 µg/L) and metallosis was not found either macroscopically or microscopically, ceramic wear was assumed to explain the sparse particles found histologically. Under high-dose cortisone therapy (30 mg prednisolone p.o. for 1 week, week 2: 25 mg, weeks 3–6: 20 mg, week 7: 15 mg, week 8: 10 mg, weeks 9–10: 5 mg) the clinical condition of the patient improved dramatically.

After 5 months of slow-reducing cortisone therapy the wound healed completely, and a total hip reimplantation with a ceramic-on-polyethylene bearing was conducted. In the clinical follow-up examination 1.5 years later there was no evidence of a recurrent arthrocele, and the patient was mobile at full load without pain.

Retrospectively, the ceramic wear volume of the primary implanted ceramic head was calculated with CAD (Figure 3, see also Supplementary data). A massive wear volume of 9.4 mm³ was determined.

**Figure 3.**
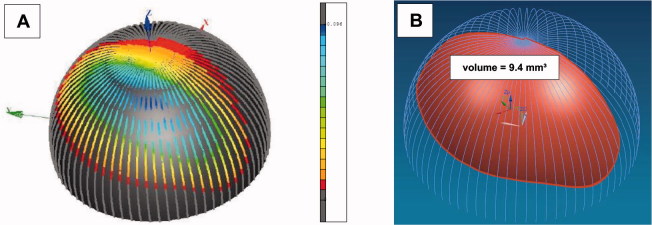
Calculation of the wear volume of the retrieved primary implanted ceramic head (A). A wear volume of 9.4 mm³ was determined (B).

## Discussion

We initially assumed PJI due to the occurrence of a sinus tract as a typical sign of an infection after the primary hip cup exchange. Different classification systems for diagnosing PJI all include a sinus tract communicating with the endoprosthesis as a sufficient criterion. When considering all our findings, PJI seems very unlikely due to the extensive and repeated negative diagnostic results, but there is a residual risk of infection, since culture-negative PJI is reported to range between 7% and 42% (Reisener et al. 2018). However, the fact that terminating the antibiotic therapy did not lead to increasing infection parameters and that temporary cortisone therapy induced a dramatical clinical improvement speak against this.

Another special feature of this case was the repeated histological finding of a granulomatous inflammation. Other non-infectious causes of granulomas such as rheumatoid arthritis and sarcoidosis were excluded by laboratory analyses. According to Krenn et al. (2014, 2016), the morphology was between an adverse reaction (SLIM type VI) and an infection (SLIM type II). Even 2 experienced pathologists could not make a conclusive, definite histological diagnosis, which emphasizes the peculiarity of this case. Granuloma formation and necrosis made SLIM classification difficult. Against the background of repeated negative microbiology with histologically proven macrophage accumulation and mild lymphocytosis, SLIM type VI appears more likely.

While light microscopy revealed only sparse wear particles, further non-routine histological examination showed intracellular particles and the wear calculation of the retrievals showed massive wear of the ceramic head. Therefore, we assume that the main trigger for the recurrent arthrocele formation was ceramic wear. It is surprising that despite massive ceramic wear only sparse particles were found histologically. However, wear-induced ceramic particles are difficult to detect by conventional histology, if at all, as the size ranges from 20 to 100 nm and only in some cases up to several micrometers (Perino et al. 2018).

The key question is whether the formation of the recurrent arthrocele was due to an adverse reaction to metal or ceramic abrasion particles. ALTR or cyst formation is reported in metal-on-metal bearings (Mabilleau et al. 2008) or due to corrosion at modular taper junctions (Gill et al. 2012). Only in a few cases was ALTR also reported in ceramic-on-polyethylene bearings, which was attributed to metal abrasion particles from the taper (Bonnaig et al. 2011) or ceramic wear particle induced abrasion of the coating of the prosthesis stem (Bohler et al. 2000). We cannot rule out that arthrocele formation was caused by an ALTR on metal abrasion particles in our case and a limitation is that we did not examine the neck taper microscopically to exclude taper abrasion. However, no macroscopic or microscopic metallosis was found and the patient’s metal ion levels were normal. Pseudotumors in patients with metal-on-metal bearings are reported to show significantly higher serum metal ion levels than those without pseudotumor formation (Kwon et al. 2011). We therefore consider it highly probable that ceramic wear led to the development of ALTR with arthrocele formation.

In vitro studies concerning the extent of a biological response of ceramic wear particles are inconsistent, but alumina particles in their clinically relevant nanometer size are supposed to have a limited impact on cell viability and cytokine production (Petit et al. 2002, Gibon et al. 2017). Alumina particles in the clinically relevant nanometer range are reported to show a cytotoxic effect on human histiocytes, although this effect was weaker than exposition with cobalt-chromium particles (Germain et al. 2003). Furthermore, macrophages showed increased phagocytosis of ceramic particles up to 2 µm in size when raising the particle concentration and macrophage viability decreased with particle size and concentration if greater than 2 µm, whereas smaller particles with 0.6 µm were only cytotoxic at higher concentrations (Catelas et al. 1998). There are few studies that have investigated the effects of ceramics on periprosthetic tissue. In vivo experiments on rats found a moderate non-specific granulomatous response of the synovial membrane after injection of ceramic particles with a size below 1 µm into the knee joint (Roualdes et al. 2010). The histological examination of our patient’s samples also revealed granulomatous changes in the (neo-)synovial tissue.

In line with our assumption, a recent case reported ceramic wear induced pseudotumor formation several years after THA with a ceramic-on-ceramic articulation (Campbell et al. 2017). Other studies revealed that ceramic wear particles mainly caused a response of macrophages in biopsies of revised hip protheses with a ceramic-on-ceramic bearing (Mochida et al. 2001). Pronounced periprosthetic tissue fibrosis was found in 9 patients with ceramic-on-ceramic articulation after total hip revision surgery compared with other bearings. The effects of ceramics were further examined by in vitro experiments, in which fibroblasts and peripheral blood mononuclear cells showed an inflammatory response when incubated on ceramic surfaces, especially on alumina-toughened zirconia (Bertrand et al. 2018). Taken together, these studies illustrate that ceramic wear may be biologically active.

**Figure 4.**
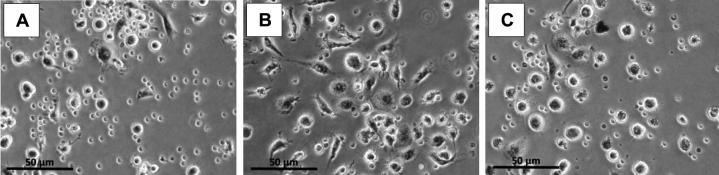
Microscopic images of the patient’s isolated peripheral blood mononuclear cells (PBMCs). (A) Before exposure of PBMCs to alumina ceramic particles. (B) After exposure of PBMCs to alumina ceramic particles, cell differentiation and adherence increased. However, cell differentiation and adherence were slower than in healthy controls (results not shown) as recently published (Klinder et al. 2018). (C) Decreased cell adherence and cell differentiation of PBMCs after increasing the alumina ceramic particle concentration.

The appearance of a sinus tract is not a sign of ALTR or has not yet been described as such. In order to examine the inflammatory response of the patient to ceramic particle exposure, we performed further analysis of the patient’s peripheral blood mononuclear cells (PBMC). In comparison with the results of healthy subjects that were recently published (Klinder et al. 2018) the patient’s isolated PBMCs showed a lower cell adherence and differentiation after exposure to alumina ceramic particles. This effect was even more pronounced with increasing particle concentration (Figure 4). The formation of a sterile sinus tract may thus be explained by an altered local immunological reaction of PBMCs to ceramic wear particles mimicking PJI.

In conclusion, ceramic wear should be considered as a differential diagnosis for recurrent arthrocele and sinus tract formation with negative microbiological cultures.

## Conflicts of interest

All authors declare that they have no conflict of interest concerning this article and report no financial support. All procedures performed in this case report involving the patient were in accordance with the ethical standards of the local ethics committee. Informed consent was obtained from the patient.

## Supplementary data

The wear volume determination method is available as supplementary data in the online version of this article, http://dx.doi.org/10.1080/17453674.2019.1616997

## Supplementary Material

Supplemental Material
