# Supplementary material for: Recurrent arthrocele and sterile sinus tract formation due to ceramic wear as a differential diagnosis of periprosthetic joint infection — a case report
Source: Acta Orthop. 2019 May 16;90(5):501–4. doi: 10.1080/17453674.2019.1616997 (PMC6746295; doi:10.1080/17453674.2019.1616997)
Supplement: Supplemental Material [file IORT_A_1616997_SM8186.pdf]

Supplementary data

We proceeded as follows to determine the wear volume of the Biolox forte head:

1. Tactile measurement (HEXAGON Leitz Reference Xi 10.7.6) with the following measurement parameters: Measurement of the surface with 72 meridians of 130 touch points each, individual meridians rotated by 5°. This results in a point cloud of approx. 10,000 measuring points.
2. Evaluation by means of false color comparison, i.e., comparison of the ACTUAL and SET geometry.
3. Volumetric determination of wear by CAD. Individual meridians were imported into the program Siemens NX 10.0.3.5 and splines were generated from them and converted into a volume model. A comparison of the ACTUAL and SET geometry is then made and the wear is output as volume (mm^3)
